# An information theoretic model of information processing in the Drosophila olfactory system: the role of inhibitory neurons for system efficiency

**DOI:** 10.3389/fncom.2013.00183

**Published:** 2013-12-20

**Authors:** Faramarz Faghihi, Christoph Kolodziejski, André Fiala, Florentin Wörgötter, Christian Tetzlaff

**Affiliations:** ^1^Department of Computational Neuroscience, Bernstein Center for Computational Neuroscience, III. Institute of Physics - Biophysics, Georg-August-UniversitätGöttingen, Germany; ^2^Molecular Neurobiology of Behavior, Johann-Friedrich-Blumenbach-Institute for Zoology and Anthropology, Georg-August-UniversitätGöttingen, Germany

**Keywords:** *Drosophila*, information theory, mutual information, system efficiency, inhibitory neurons, odor concentration

## Abstract

Fruit flies (*Drosophila melanogaster*) rely on their olfactory system to process environmental information. This information has to be transmitted without system-relevant loss by the olfactory system to deeper brain areas for learning. Here we study the role of several parameters of the fly's olfactory system and the environment and how they influence olfactory information transmission. We have designed an abstract model of the antennal lobe, the mushroom body and the inhibitory circuitry. Mutual information between the olfactory environment, simulated in terms of different odor concentrations, and a sub-population of intrinsic mushroom body neurons (Kenyon cells) was calculated to quantify the efficiency of information transmission. With this method we study, on the one hand, the effect of different connectivity rates between olfactory projection neurons and firing thresholds of Kenyon cells. On the other hand, we analyze the influence of inhibition on mutual information between environment and mushroom body. Our simulations show an expected linear relation between the connectivity rate between the antennal lobe and the mushroom body and firing threshold of the Kenyon cells to obtain maximum mutual information for both low and high odor concentrations. However, contradicting all-day experiences, high odor concentrations cause a drastic, and unrealistic, decrease in mutual information for all connectivity rates compared to low concentration. But when inhibition on the mushroom body is included, mutual information remains at high levels independent of other system parameters. This finding points to a pivotal role of inhibition in fly information processing without which the system efficiency will be substantially reduced.

## 1. Introduction

The olfactory system of insects is essential for their search for food and mates. Structures and neuronal circuits have evolved to detect, amplify and discriminate weak odor signals in fluctuating sensory environments in which the animals receive much higher odor concentrations at the odor source as compared to the concentration present at initial odor detection. Due to this complex situation, the structure and function of the olfactory system of *Drosophila melanogaster* has been studied for many years (Vosshall and Stocker, 2007; Wilson, 2013).

**Figure 1:**
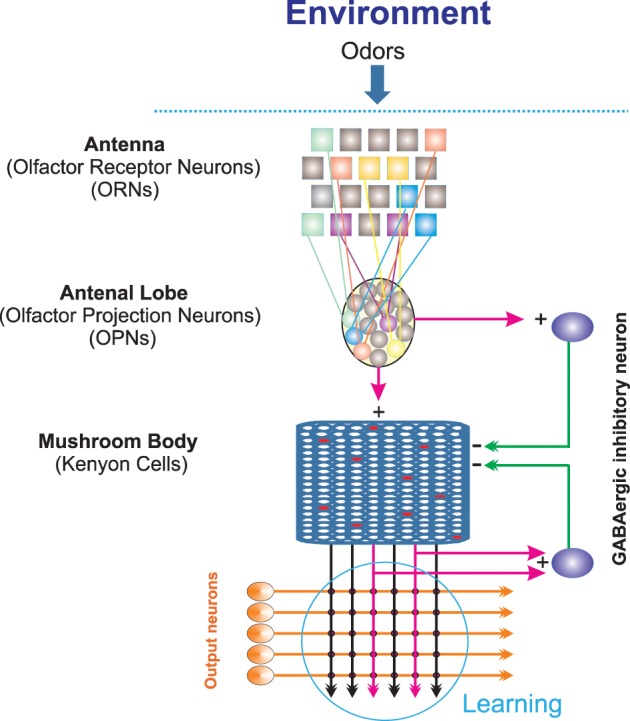
**Schematic illustration of the Drosophila olfactory system**. Odor stimuli from the environment bind to the receptors located on the antenna; each olfactory receptor neuron expresses one specific type of receptor (illustrated by different colors). Olfactory receptor neurons of the same class project to one glomeruli of the antennal lobe. Projection neurons in the antennal lobe, in turn, activate Kenyon cells in the mushroom body and lateral horn by cholinergic, excitatory synapses. GABAergic, inhibitory neurons projecting from the lateral horn and from the antennal lobes to the mushroom body calyx provide inhibitory effects on activated Kenyon cells. The synapses between output neurons and Kenyon cells is the area where learning takes place. Thus, for optimal learning this area needs information transferred from the environment to this site.

In the following we provide a brief overview about the basic parts of *Drosophila* olfactory system (see also Figure 1). The olfactory information about an odor can be characterized by quality (chemical compounds in a given odor), quantity (concentration), spatial distribution, and temporal fluctuation (Laurent, 2002). An odorant in the environment binds to olfactory receptors present on the membrane of olfactory receptor neurons (ORNs) which are located on the third segments of the fly's antenna and on the maxillary palps (Vosshall and Stocker, 2007). Typically, each of these ORNs expresses only one specific olfactory receptor. The binding of odorants to receptors can lead to either excitation or inhibition of the respective ORN. The ORNs axonal projections bundle together as tracts and transfer the information to the antennal lobe, the primary olfactory neuropil of the insect brain. The antennal lobe is composed of 50 discrete spherical neuropil regions called glomeruli, where ORNs synapse onto either local interneurons or olfactory projection neurons (OPNs). As a principle, ORNs expressing the same specific olfactory receptor project into the same glomeruli. As a result, odors are mapped in the antennal lobe in terms of combinatorial patterns of glomerular activity (Fiala et al., 2002). The odor information is transmitted from the antennal lobe to the mushroom body and the lateral horn. In the mushroom body information is represented in a sparse code (Honegger et al., 2011). Several parameters influence this sparse code as, for instance, the connectivity rate between the antennal lobe and the mushroom body or the firing threshold of Kenyon cells (Turner et al., 2008; Luo et al., 2010; Caron et al., 2013).

In *Drosophila*, the mushroom body (Kenyon cells) receives olfactory signals from the antennal lobe (OPNs) at the calyx, the major dendritic input side of the mushroom body. The connectivity rate between OPNs and Kenyon cells, i.e., how many of about 150 OPNs synapse onto how many of the Kenyon cells, has been estimated to be relatively low (about 0.3; Perez-Orive et al., 2002; Honegger et al., 2011). A number of different mushroom body neurons, the output neurons, are likely to be involved in olfactory learning and memory. Therefore, the mushroom body is believed to be critical for associative olfactory learning (Heisenberg, 2003; Fiala, 2007). This implies that the olfactory information of the environment has to be transmitted efficiently via the antenna and antennal lobe to the mushroom body to guarantee adequate learning.

In this work, the transmission efficiency is measured by an information theoretic measure often used in neuroscience called “mutual information” (Borst and Theunissen, 1999; Dimitrov et al., 2011). This allows us to study the role of connectivity rate and firing threshold under different environmental conditions. Amongst others, we see a behaviorally unexpected result—with higher odor concentration less information is transmitted to the mushroom body.

However, work on larger insects (e.g., Locust) has revealed that the mushroom body receives via lateral horn feed-forward inhibition from the antennal lobe (Gupta and Stopfer, 2012) and, furthermore, feedback inhibition from the mushroom body itself (Gru, 1999). Thus, we extended our model by different strengths of inhibition to analyze its influence on information transmission. This way we can show that for a broad regime of inhibitory strengths the transmission efficiency becomes optimized for high odor concentrations.

## 2. Materials and methods

In this work we analyze how much environmental information the *Drosophila* olfactory system transmits to the mushroom body and, hence, to the output neurons. Several physiological parameters influence this transmission in different ways. Here, we determine the influence of the connectivity rate between OPNs and Kenyon cells and the firing threshold of the Kenyon cells. Specifically, the impact of different strengths of inhibiting Kenyon cells firing is assessed. To quantify the transmission efficiency, we calculated the mutual information for the different conditions between the environmental input and the mushroom body output.

In the following, we present the structure of the model, the implementation of inhibition, and the way of calculation mutual information.

### 2.1. Structure of the model

**Figure 2:**
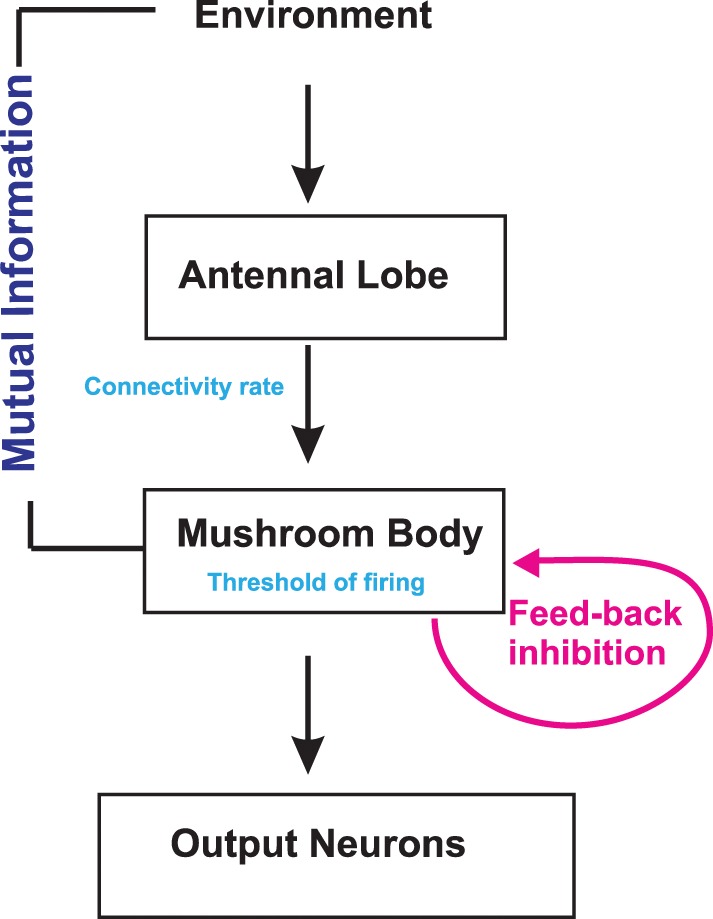
**Schematic illustration of the model structure**. Odor information in the environment is sent via the antenna to the antennal lobe and subsequently to the mushroom body. Inhibitory neurons contribute to the information processing. There are two known inhibitory connections to the mushroom body calyx originating either from the lateral horn or from the mushroom body itself. Mutual Information between environment and Kenyon cells is measured for different activation threshold, connectivity rates, and inhibition strengths.

The simplified anatomical structure used in this model (Figure 2) includes two neural circuits with the antenna and the antennal lobe as one circuit and the mushroom body as the second one. We combined the antenna and the antennal lobe in a circuit because the ORNs, which express the same specific olfactory receptors, send their axons to the same glomeruli, and OPNs in each glomerulus receive information just from ORNs of the same class. Reported exceptions from these rules, i.e., ORNs expressing more than one receptor, ORNs targeting more than one glomerulus and multiglomerular OPNs receiving information from many glomeruli, are disregarded for simplicity. Furthermore, we assume that each of the 50 glomeruli (Caron et al., 2013) consists of one OPN. Thus, in our model, an odor *k* presented to the circuit reaches a subset of glomeruli or OPNs as odors consists of several different chemicals. These diverse odor structures yield different numbers of reached OPNs *N*^*k*^_*OPN*_. These OPNs are the subgroup which can be activated by the odor *k*. Their number is drawn from a Gaussian distribution with mean μ_*OPN*_ = 35 and variance σ^2^_*OPN*_ = 8. This group of OPNs, in turn, projects to a subgroup of Kenyon cells in the mushroom body. As we show below, the exact number of Kenyon cells *N*_*KC*_ does not alter the results qualitatively.

As described before, each odor *k* presented to the system activates a subgroup of neurons in the antennal lobe. Each OPN *j* can be either active χ^*k*^_*j*_ = 1 or silent χ^*k*^_*j*_ = 0. The number of activated OPNs depends on the concentration *c*^*k*^_*odor*_ of the odor. Low concentrations (*c*^*k*^_*odor*_ ≈ 0) activate a few, high concentrations (*c*^*k*^_*odor*_ ≈ 1) activate nearly all reached OPNs. However, due to noise, this number can vary over trials. Thus, the activation of each reached OPN is probabilistic dependent on the following equation:

(1) $$p_{OPN}^{k}=1-e^{-\beta \cdot c_{odor}^{k}}.$$

The parameter β determines the relation between odor concentration and neuronal activity and, without loss of generality, is set equal to 1.32. Thus, each odor is represented in the antennal lobe as a state of 1s and 0s of length *N*^*k*^_*OPN*_. This state is transmitted via the synapses in the calyx to the Kenyon cells. Thereby, each OPN *j* connects to Kenyon cell *i* with probability *r* (drawn from the interval from zero to one without boundaries) which defines the connectivity rate. Thus, the connection *c*_*i,j*_ is either zero or one depending on *r*. All *N*_*KC*_ Kenyon cells are modeled as simple integrate-and-fire neurons with firing threshold Θ (between 1 and 20):

(2) $$\psi_{i}^{k}=\{\begin{array}{ll} 1 & \text{if}\sum_{j}^{N_{OPN}^{k}}c_{i,j}\cdot \chi_{j}^{k}>\Theta \\ 0 & \text{if}\sum_{j}^{N_{OPN}^{k}}c_{i,j}\cdot \chi_{j}^{k}\leq \Theta . \end{array}$$

Thus, each odor yields via the antenna and antennal lobe to a specific firing pattern of Kenyon cells in the mushroom body.

### 2.2. Inhibition

To assess the influence of different inhibitory strengths on mushroom body, we introduce a probability *p*^*k*^_*I*_ that Kenyon cells firing can be inhibited. This probability depends on the average activity in the mushroom body ${\tilde{\chi }}^{k}$ without inhibition.

(3) $$p_{I}^{k}=e^{-\frac{\alpha }{{\tilde{\chi }}^{k}}}$$

**Figure 3:**
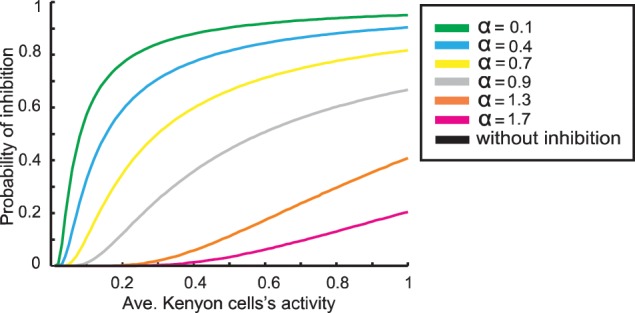
**The relation between average Kenyon cells activity and probability of feedback inhibition for different parameter values α**. Note, a higher value of α induces less inhibition.

The parameter α (between 0.05 and 1.75) determines the different strengths of inhibition (Figure 3). Thus, we first derive the activity of the Kenyon cells as described before, calculate the probability of inhibiting each Kenyon cell spike, and derive the new Kenyon cell firing. This new state, dependent on the inhibitory feedback, is the final output of the mushroom body.

### 2.3. Mutual information

From an information theoretic point of view the *Drosophila melongaster* olfactory system can be considered as an “information channel” which transmits environmental information to the mushroom body. The efficiency of such a channel can be characterized by “mutual information.” To calculate mutual information between environment (odors) and mushroom body (Kenyon cells) we need to assess the probability distribution of Kenyon cells firing *p*(*n*). Each *n* represents one out of 𝔑 = 2^*N*^_*KC*_states of neuronal activity in the mushroom body. The distribution of Kenyon cells firing depends on the information from the environment. This information is represented by the probability distribution *p*(*k*) of 𝔎 odors presented to the olfactory system. In the following we assume that these odors are equiprobable. Thus, the mutual information between environment and mushroom body is

(4) $$MI=\sum_{n=1}^{𝔑}\sum_{k=1}^{𝔎}p(n,k)\log\left(\frac{p(n,k)}{p(n)p(k)}\right).$$

*p*(*n, k*) is the joint probability distribution of Kenyon cells states and presented odors. Note, to guarantee calculability we set *p*(*n*) = 10^−8^ when state *n* does not occur.

### 2.4. Experimental procedure

In this study, we tested the efficiency of information transmission dependent on several physiological and environmental parameters as average connectivity *r* between antennal lobe and mushroom body, firing threshold Θ of Kenyon cells, inhibition (parameter α), number of Kenyon cells *N*_*KC*_, and odor concentration *c*_*odor*_. As the actual connectivity is created probabilistically (see before), we tested 20 different systems (“flies”) with each having another connectivity for one set of Θ, *r*, α, *c*_*odor*_, and *N*_*KC*_. Each system was tested in 100 trials with 100 different odors of same concentration. The odors are specified by the number of OPNs reached. In every trial a different subset of the reached OPNs becomes active.

Since the 100 trials do not provide sufficient statistics to assess the joint probability distribution *p*(*n, k*) (how often which state *n* occurs given odor *k*) and the probability distribution of Kenyon cells firing *p*(*n*) (how often each state *n* occurs in total) we used the “Quadratic Extrapolation procedure” to correct for the sampling bias problem (Panzeri et al., 2007). This procedure assumes that the biased mutual information *MI*_uncorrected_ can be approximated as second order expansion in $\frac{1}{N}$, where *N* is the number of trials. That is

(5) $$MI_{corrected}=MI_{uncorrected}-\left(\frac{a}{N}+\frac{b}{N^{2}}\right)$$

where a, b are free parameters that are estimated from fractions ($\frac{N}{2}$ and $\frac{N}{4}$) of the data and MI_corrected_ is the true calculated mutual information. Then, for each “fly” we calculate the mutual information and average over them.

## 3. Results

### 3.1. Information transmission under various conditions without inhibition

In the following we will show the results without inhibition on Kenyon cells and mainly demonstrate that this leads to undesired and unrealistic characteristics. The value of the firing threshold of Kenyon cells in real cells is unknown. Therefore, all possible thresholds from 1 to 20 were studied. Similarly, little information about the exact connectivity between OPNs and Kenyon cells exists but studies have shown that this connectivity is small (Perez-Orive et al., 2002; Honegger et al., 2011). Thus, we also tested the system with different connectivity rates. Hence, mutual information between environment and 10 or 20 Kenyon cells, respectively, was measured for all combinations of connectivity rate and firing threshold at low (*c*^*low*^_*odor*_ = 0.15) as well as high (*c*^*high*^_*odor*_ = 0.75) odor concentrations. First, for all results in this section it is assumed that there is no inhibition on the Kenyon cells. In the next section we investigate the inhibitory influences, too.

**Figure 4:**
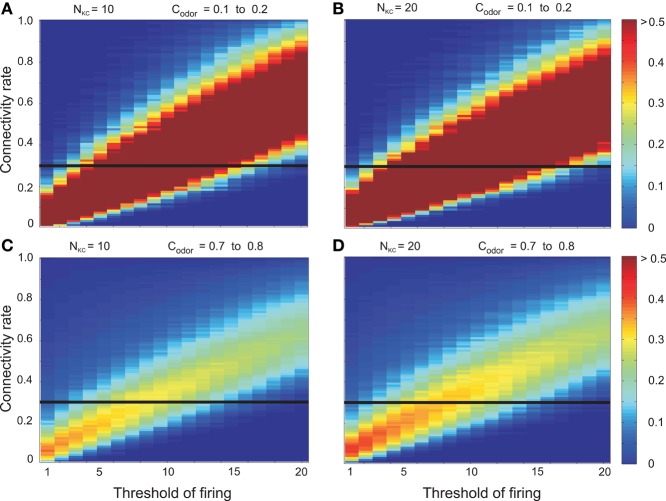
**Mutual information between environment (here 5 odors) and Kenyon cells for different firing thresholds of Kenyon cells and connectivity rate between the antennal lobe and the mushroom body in absence of inhibition on mushroom body**. For each connectivity rate there is one unique threshold that corresponds to maximum mutual information. Moreover, increasing the threshold requires a higher connectivity rate to obtain maximum mutual information (thresholds between 1 and 20). The number of Kenyon cells does not alter the results qualitatively. **(A,B)** Low odor concentration (*c*^*low*^_*odor*_ = 0.15). **(C,D)** High odor concentration (*c*^*high*^_*odor*_ = 0.75): for each connectivity rate and threshold of firing, mutual information is decreased compared to low odor concentration. Parameters: **(A)** *N*_*KC*_ = 10, *c*^*low*^_*odor*_ = 0.15 **(B)** *N*_*KC*_ = 20, *c*^*low*^_*odor*_ = 0.15 **(C)** *N*_*KC*_ = 10, *c*^*high*^_*odor*_ = 0.75 **(D)** *N*_*KC*_ = 20, *c*^*high*^_*odor*_ = 0.75. The horizontal lines indicate the connectivity rate equal to 0.3 (shown in this figure).

Figures 4A,B shows the dependency of mutual information for 10 as well as 20 Kenyon cells on the threshold and connectivity rate at low odor concentration (*c*^*k*^_*odor*_ = *c*^*low*^_*odor*_ for all odors *k*). For each threshold there is one unique connectivity that corresponds to maximum mutual information. Another observation is the presence of an almost linear relation between connectivity rate and threshold for the maximal mutual information. Comparing the results for 10 and 20 Kenyon cells shows that the number of Kenyon cells does not qualitatively alter the results. A qualitatively similar behavior is observed for high odor concentration (*c*^*k*^_*odor*_ = *c*^*high*^_*odor*_). Hence, for each connectivity rate there is a shift to the right of the firing threshold that corresponds to maximum mutual information (Figures 4C,D).

**Figure 5:**
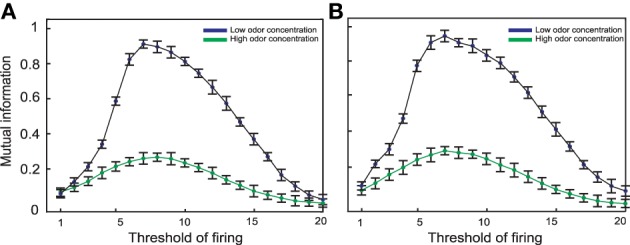
**Mutual information between the environment and (A) 10 Kenyon cells or (B) 20 Kenyon cells for different thresholds**. The connectivity rate set to 0.3 for both low and high odor concentrations. The maximum mutual information is obtained at threshold equal to 7 for low concentration and 8 when high odor concentration was presented to the neural system. For both levels of concentrations mutual information is decreased by increasing threshold. This clearly shows that an increase in odor concentration in absence of inhibition to Kenyon cells leads to a dramatic decrease in mutual information.

As a low connectivity rate between antennal lobe and mushroom body (*r* ≈ 0.3; Butcher et al., 2012; Caron et al., 2013) is assumed in flies, we show a cross-section of Figure 4 (Figure 5). The results clearly show a decrease in mutual information for high odor concentration and also a shift to higher threshold to obtain maximum mutual information as the firing rate becomes too high to enable a proper discrimination between odors. All these observations are in conflict with behavioral experiments where higher odor concentrations increase learning and memory efficiency, suggesting highly efficient information processing. Little is known with respect to plasticity of Kenyon cells and, thus, effective connectivity in this system might not change. The firing threshold, on the other hand, might well be a controllable parameter helping the insect when it encounters environments with different odor concentrations. However, such candidate mechanisms seems to be too slow (e.g., intrinsic plasticity; Triesch, 2007; Turrigiano, 2011) compared to behavioral time scales. Another option to control the efficiency of information transmission in a fluctuating odor concentration is the inhibition on Kenyon cells which will be investigated next.

### 3.2. The role of inhibition on mutual information

In the previous section we showed that the efficiency of information transmission depends critically on the odor concentration. However, the GABAergic inhibitory effect on Kenyon cells seems to have a critical role in sparse coding (Assisi et al., 2007). As sparse coding is related to mutual information, we next analyze the role of inhibition on Kenyon cells in conjunction with the information transfer from the environment to the olfactory learning area in *Drosophila*.

**Figure 6:**
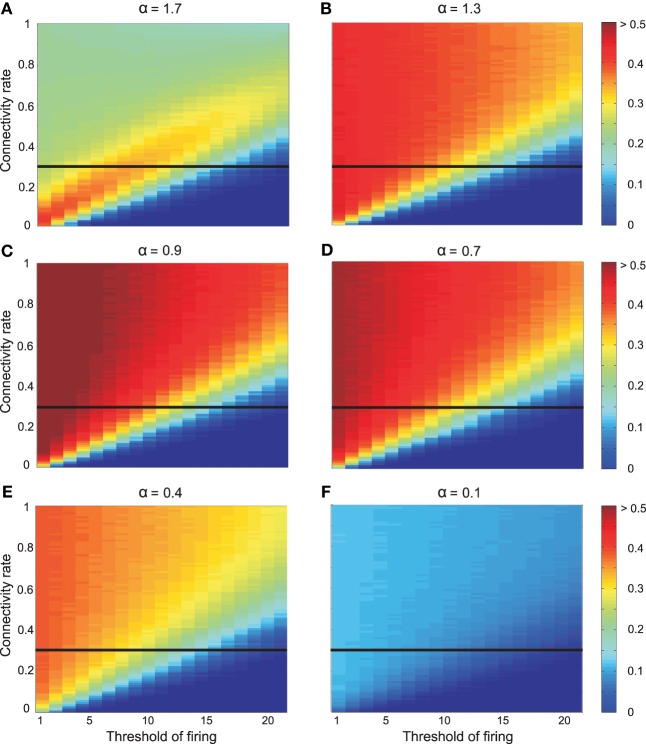
**Mutual information between environment (5 odors) and 10 Kenyon cells with the effect of inhibition on Kenyon cells and high odor concentration *c*^*high*^_*odor*_ = 0.75**. The inhibition parameter α is equal to **(A)** 1.7, **(B)** 1.3, **(C)** 0.9, **(D)** 0.7, **(E)** 0.4, and **(F)** 0.1. Different parameter values yield different results on mutual information. The horizontal line shows the connectivity rate equal to 0.3 (shown in Figure 7).

**Figure 7:**
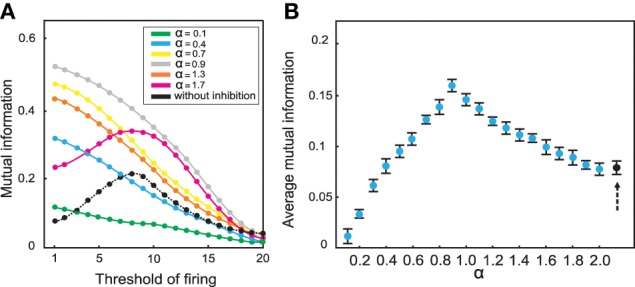
**Mutual information between mushroom body and environment for connectivity rate equal to 0.3 for high odor concentration**. **(A)** The mutual information is for most strengths of inhibition larger than without inhibition (dashed line). **(B)** Comparing the average mutual information with and without inhibition (black dot) shows that for most α-values mutual information is higher than mutual information without inhibition.

Inhibition leads to an entirely different picture (Figure 6): Figure 6 illustrates the effect of inhibition on mutual information for different connectivity rates and thresholds when high odor concentration was presented to the system (*N*_*KC*_ = 10). Different strengths of inhibition (different α values) result to different levels of mutual information for different connectivity rates and thresholds. At connectivity rate equal to 0.3 (Figure 7), most of the cases with inhibition are more effective in information transmission (higher mutual information) than without inhibition (dashed line). However, too strong inhibition leads to the effect that activities drop dramatically and, therefore, information transmission is decreased.

**Figure 8:**
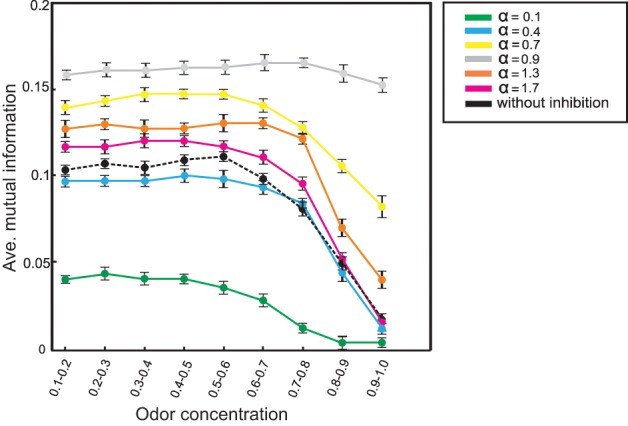
**Mutual information between mushroom body (*N*_*KC*_ = 10) and environment at connectivity rate equal to 0.3 and averaged over thresholds (1–20)**. Different odor concentrations were presented to the neural system with (α = 0.1 to 1.7) and without inhibition. Comparing mutual information for different α parameter values demonstrate that feedback inhibition increases mutual information for all concentrations. Furthermore, for several α-values mutual information does not show such a dramatic drop in performance for increasing odor concentration.

Inhibition increases mutual information not only for one high odor concentration value: Figure 8 shows the effect of inhibition on information transmission over different odor concentrations (averaged over all thresholds and *r* = 0.3). For all concentration values the system with different inhibition strengths performs significantly better than without inhibition. Furthermore, the system does not show such a dramatic drop in information transmission increasing odor concentration. Thus, the olfactory system with feedback inhibition guarantees all the time an effective transmission of environmental information to the learning area when the animal moves toward the source of odor.

## 4. Discussion

Information theory has helped neuroscientists to study some structural and functional parameters that are difficult to assess by experiments and it offers some measures to evaluate information transfer by neural systems (Borst and Theunissen, 1999; Dimitrov et al., 2011). Furthermore, some tools from information theory are used to measure how much information a neural response contains about the stimulus in the environment and the statistical significance of variation in neural response for different stimulus intensities. The first attempts to apply information theory in neuroscience were to measure neural information flow in neural systems, as well as, the constraints that information theory imposes on the capability of neural system for communication. Thus, information theoretic measures have been used to infer the functional connectivity in neural systems (Singh and Lesica, 2010; So et al., 2011). Another step was to discuss information as a constraint on neural system structure and function to optimize information transmission (Attneave, 1954). This approach that the optimal information transfer guides the neuronal structure is still a very active field in neuroscience (Shlens et al., 2009; Vanni and Rosenström, 2011).

In the current study, we have analyzed with tools from information theory the influence of olfactory system's parameter on information transmission between environment and mushroom body. The abstract model proposed in this study is capable of considering connectivity rate between antennal lobe and mushroom body, threshold of firing of neurons in the mushroom body, as well as the role of inhibition in different environmental situations that *Drosophila melanogaster* may encounter through its life time. For this purpose, we calculated mutual information between the environment composed of a set of odors with different levels of odor concentrations and a subpopulation of neurons in the mushroom body. We found that a plain feed-forward system produces undesirable effects, like a drop in mutual information for increasing odor concentration. This is in contrast to behavioral studies (Masek and Heisenberg, 2008; Yarali et al., 2009) which have demonstrated that higher odor concentration does not lead to a decrease in *Drosophila* efficiency for learning, memorizing, and discrimination of odors. Therefore, one would expect that the measure mutual information used in this study should be able to describe this phenomenon and, furthermore, predict the conditions necessary to obtain highest system efficiency. In absence of feedback inhibition, our simulations clearly assign a remarkable decrease in system efficiency for odors with high concentrations. However, feedback inhibition of different strengths helps the system to obtain high system efficiency. Interestingly, a certain strength of inhibition (here equals to 0.9) results to the best performance. This inhibition strength guarantees a good system performance independent of the odor concentration. Hence, a pharmacological manipulation of this parameter *in vivo* should result to measurable changes in fly's behavior.

These findings support the idea of a key role of inhibition in keeping the system at or close to maximal mutual information or information transfer for all Kenyon cells (with varying connectivity rate) when the fly navigates in its natural environment, where odor concentration can very strongly vary. For instance, sensing very low odor concentration from a far distance navigating to it and eventually finding the source of odor (food or mate, for example) where the concentration has increased thousandfold. The importance of high and stable mutual information between a dynamic environment and the Kenyon cells becomes even clearer if we consider that the synapses between Kenyon cells and output neurons are very likely the place of association based learning (Séjourné et al., 2011).

### Conflict of interest statement

The authors declare that the research was conducted in the absence of any commercial or financial relationships that could be construed as a potential conflict of interest.
